# The Quality of Short Videos as a Source of Coronary Heart Disease Information on TikTok: Cross-Sectional Study

**DOI:** 10.2196/51513

**Published:** 2024-09-03

**Authors:** Xun Gong, Meijuan Chen, Lihong Ning, Lingzhong Zeng, Bo Dong

**Affiliations:** 1 Department of Cardiology and Cardiac Rehabilitation Center Hunan Provincial People's Hospital (The First Affiliated Hospital of Hunan Normal University) Changsha China

**Keywords:** coronary heart disease, content quality, social media, short-video platform, TikTok

## Abstract

**Background:**

Coronary heart disease (CHD) is a leading cause of death worldwide and imposes a significant economic burden. TikTok has risen as a favored platform within the social media sphere for disseminating CHD-related information and stands as a pivotal resource for patients seeking knowledge about CHD. However, the quality of such content on TikTok remains largely unexplored.

**Objective:**

This study aims to assess the quality of information conveyed in TikTok CHD-related videos.

**Methods:**

A comprehensive cross-sectional study was undertaken on TikTok videos related to CHD. The sources of the videos were identified and analyzed. The comprehensiveness of content was assessed through 6 questions addressing the definition, signs and symptoms, risk factors, evaluation, management, and outcomes. The quality of the videos was assessed using 3 standardized evaluative instruments: DISCERN, the Journal of the American Medical Association (JAMA) benchmarks, and the Global Quality Scale (GQS). Furthermore, correlative analyses between video quality and characteristics of the uploaders and the videos themselves were conducted.

**Results:**

The search yielded 145 CHD-related videos from TikTok, predominantly uploaded by health professionals (n=128, 88.3%), followed by news agencies (n=6, 4.1%), nonprofit organizations (n=10, 6.9%), and for-profit organizations (n=1, 0.7%). Content comprehensiveness achieved a median score of 3 (IQR 2-4). Median values for the DISCERN, JAMA, and GQS evaluations across all videos stood at 27 (IQR 24-32), 2 (IQR 2-2), and 2 (IQR 2-3), respectively. Videos from health professionals and nonprofit organizations attained significantly superior JAMA scores in comparison to those of news agencies (*P<*.001 and *P*=.02, respectively), whereas GQS scores for videos from health professionals were also notably higher than those from news agencies (*P*=.048). Within health professionals, cardiologists demonstrated discernibly enhanced performance over noncardiologists in both DISCERN and GQS assessments (*P*=.02). Correlative analyses unveiled positive correlations between video quality and uploader metrics, encompassing the positive correlations between the number of followers; total likes; average likes per video; and established quality indices such as DISCERN, JAMA, or GQS scores. Similar investigations relating to video attributes showed correlations between user engagement factors—likes, comments, collections, shares—and the aforementioned quality indicators. In contrast, a negative correlation emerged between the number of days since upload and quality indices, while a longer video duration corresponded positively with higher DISCERN and GQS scores.

**Conclusions:**

The quality of the videos was generally poor, with significant disparities based on source category. The content comprehensiveness coverage proved insufficient, casting doubts on the reliability and quality of the information relayed through these videos. Among health professionals, video contributions from cardiologists exhibited superior quality compared to noncardiologists. As TikTok’s role in health information dissemination expands, ensuring accurate and reliable content is crucial to better meet patients’ needs for CHD information that conventional health education fails to fulfill.

## Introduction

Coronary heart disease (CHD) is a condition that poses a significant threat to human health and is characterized by high rates of morbidity, mortality, and disability [1]. Worldwide, an estimated 254.2 million people live with CHD, while approximately 9.21 million deaths were attributed to the condition [2]. Within the United States, around 20.5 million inhabitants aged 20 years or older have CHD. The overall prevalence reaches 7.1% among the US population over the age of 20 years [2]. Notably, both the prevalence and mortality rate of CHD in China are also disconcerting. For Chinese citizens aged 15 years and older, the prevalence was 10.2 per 1000, and 27.8 per 1000 among those over 60 years in 2013. The estimated patient count hits were 11.39 million, with urban and rural mortality rates at 121.59 and 130.14 per 100,000, respectively. CHD ranks as a predominant cause of mortality worldwide and imposes a substantial economic burden [1]. In China, the total hospitalization costs for CHD soared to RMB 125.625 billion (approximately US $18.45 billion at a 6.8:1 RMB to US $ exchange rate) in 2019, exerting a hefty financial toll on the nation, communities, and families [3]. The burgeoning prevalence of CHD is propelled by factors such as aging, hypertension, diabetes, hyperlipidemia, obesity, smoking, and lack of exercise [1]. Consequently, there exists a pressing need for medical services among patients with CHD, yet the current deficit in health care resources obstructs the fulfillment of their consultation and treatment necessities [4].

Obtaining medical services for CHD is a challenging and time-consuming process. Patients must navigate a series of procedures including booking appointments, enduring long waits for consultations, and undergoing examinations and treatments in large-scale hospitals. For instance, within the Spanish National Health System, the mean waiting time before seeing a general practitioner stands at 3.36 days, escalating to 88.03 days for specialist consultations [5]. Moreover, the hectic schedules of medical professionals often lead to cursory attention being given to comprehensive information consultations and patient education. It is noteworthy that a considerable majority (89%) of hospitalized patients with CHD receive some form of health education prior to discharge. Nonetheless, there is a clear and persistent need for health information at discharge, extending 6 months thereafter [6]. Given the brief nature of hospital stays and suboptimal participation in cardiac rehabilitation initiatives, those with severe CHD—particularly post–percutaneous coronary intervention—exhibit limited health literacy [7]. The escalating appetite for CHD-related information among patients, coupled with the inherent limits of the existing health care paradigm, necessitates innovative methodologies to bridge this gap. Presently, the proliferation of smartphones and advancements in informational technology have facilitated the rise of short video platforms, which promulgate vital disease-related knowledge to patients in need [8], while social media gradually usurps traditional channels as the principal source for both personal and public health information [9]. In practice, platforms like TikTok emerge as the most approachable means for those with CHD to secure critical health information, thereby significantly enhancing their comprehension, potentially bettering adherence to therapeutic regimens, and improving overall outcomes.

Accessing disease information via these platforms confers notable benefits for patients [10]. First, information accessibility transcends geographical and temporal boundaries, enabling patients to access data without the constraints of travel or operational hours of medical facilities. Second, the plethora of information sources helps to alleviate skepticism toward a singular informational outlet. Unlike visits to hospitals where consultations are limited to individual doctors, these platforms host a multitude of contributors (often health care professionals), offering diverse perspectives and insights. This plurality serves to validate the information gleaned from hospital engagements, reducing mistrust between physicians and patients arising from the paucity of information sources [11]. Third, leveraging short videos for information acquisition proves to be cost-efficient. Typically available at no cost, these contrast starkly with the considerable financial outlays associated with hospital-based treatment, encompassing medical bills, transport, accommodation, dining, and temporal expenditures [12]. Finally, to a degree, these platforms safeguard patient confidentiality. Hospital visits bear potential privacy risks [13], whereas engaging with short videos circumvents such risks and minimizes the necessity for physical interactions, a particularly salient advantage amid the COVID-19 pandemic [14]. Hence, short video platforms have emerged as pivotal repositories of disease-related information for the public. The trend toward web-based health information procurement is on the rise, with a significant number of individuals turning to internet resources to supplement their health care decision-making processes [15]. While studies affirm that judicious and efficacious use of social media can positively influence health outcomes [16], the pervasive dissemination of misleading content and subpar medical videos can dismiss these benefits [17]. For instance, an analysis of a COVID-19–related Twitter dataset from South Africa, spanning from November 8, 2020, to July 19, 2021, found that out of 976,087 tweets, 329,107 were deemed “false” using a LightGBM classifier [18]. This accentuates the issue of information quality on social platforms, underscoring the imperative of educating audiences on discerning information quality to curb the detrimental effects of misinformation. TikTok, reigning as the preeminent short video platform globally, also hosts an extensive array of patient-oriented content [19]. According to the *TikTok Health Science Data Report*, health content ranks among the top categories engaged by users [20]. As of March 2023, TikTok boasts over 35,000 verified health care providers, contributing 21,000 new entries daily, with health-related posts amassing more than 200 million views daily. Collectively, these professionals have created 4.43 million pieces of content. The *2023 TikTok Health Lifestyle New Paradigm White Paper* reveals that in the first half of 2023 alone, over 100 million health videos were uploaded, generating nearly 500 billion views [21]. With a user base exceeding 100 million regularly interacting with health content, TikTok has positioned itself as a formidable force in the digital medical sphere, second only to traditional hospital settings. The extensive dissemination of health information and substantial viewership on TikTok significantly influence patients’ knowledge levels of the diseases, thereby impacting their health care–seeking behaviors and treatment efficacies [22]. Nonetheless, the quality of disease-related videos on TikTok is inconsistent, challenging patients to identify trustworthy content and heightening the risk of misinformation. Specifically, TikTok content addressing chronic obstructive pulmonary disease, diabetes, gallstones, and inflammatory bowel disease often suffers from low quality and lacks comprehensive treatment information [23-26]. Given the higher morbidity and mortality rates associated with CHD relative to the diseases aforementioned, a considerable patient demographic turns to TikTok for CHD-related information. Yet, the quality of CHD-related content on TikTok remains unexamined, spotlighting the urgent need for quality assessment. Accordingly, this study aims to assess the quality of CHD-related videos on TikTok, providing precise guidance for both patients and content creators on the platform.

## Methods

### Ethical Considerations

This study did not encompass the use of clinical datasets, biological specimens, or nonhuman vertebrates. All data harnessed for this analysis were derived exclusively from publicly available TikTok videos, thereby safeguarding individual privacy. Moreover, the research protocol abstained from any direct engagement with platform users, obviating the need for ethical oversight or formal trial registration. All data are deidentified, and there is no identification of individual users, videos, or screenshots in this paper or its supplementary materials. Furthermore, the full dataset of posts is available to researchers upon reasonable request to the first author or the corresponding author.

### Search Strategy and Data Extraction

**Figure 1:**
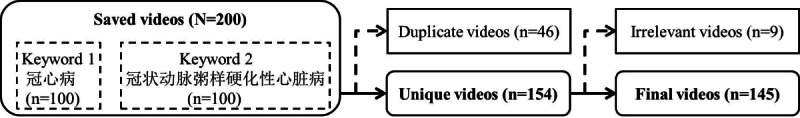
Video search strategy and screening procedure.

To mitigate bias stemming from personal recommendation algorithms, we used 3 tactics: the creation of new TikTok accounts specifically for evaluation purposes, the deactivation of personalized recommendations, and the prohibition of access to mobile location services. To identify pertinent CHD-related content on TikTok, we used 2 Chinese keywords: “冠心病” (coronary heart disease) and “冠状动脉粥样硬化性心脏病” (coronary atherosclerotic heart disease). The rationale behind selecting these keywords hinges on the fact that “coronary heart disease” is precisely defined as “coronary atherosclerotic heart disease,” thus they are accurate equivalents. In contrast, “myocardial ischemia” and “ischemic heart disease” do not correspond as exact synonyms for “coronary heart disease.” Additionally, a query for “coronary heart disease” using Baidu, China’s preeminent search engine, invariably directs to top results synonymous with coronary atherosclerotic heart disease. Video search analysis indicates an abundance of duplicates for these terms alongside scant unrelated findings, implying that TikTok’s algorithm robustly correlates “coronary heart disease” with “coronary atherosclerotic heart disease.” Such linkage fortifies the extraction of relevant footage, curtailing potential confusion from nebulous content that might skew the study’s accuracy. TikTok’s search feature offers 3 sorting methodologies: “overall ranking,” “most recent,” and “most likes,” with the default recommendation being the overall ranking—a composite that encompasses the latter 2 criteria. As most patrons adhere to this preset, our approach used the overall ranking filter to capture the foremost 100 entries from August 29, 2022, to September 2, 2022, corresponding to each keyword. This process yielded an aggregate of 200 clips. We settled on the capstone of 100 videos for a pair of reasons. First, TikTok’s algorithm prioritizes topics by relevance, propelling the most germane CHD recordings to the forefront of its listing—a sequence diluted beyond the 100th entry. Second, the general proclivity for web-based health information consumption aligns with the “principle of least effort,” wherein individuals prioritize initial returns over exhaustive examination [27]. To distill the most pertinent compilations, we excluded duplicates (n=46) and unrelated materials (n=9). Our final compendium encompassed 145 videos subject to detailed assessments (Figure 1). Using Microsoft Excel, we meticulously cataloged each video’s metadata, encapsulating descriptors, uploader account profiles (total followers, total likes, total videos, and average likes per video), and specific attributes of the curated videos (date of publication, duration in seconds, number of likes, comments, collections, and shares).

### Classification of Videos

The videos were classified according to their sources [23]: (1) health professionals, (2) news agencies (such as network media, newspapers, television, and radio), (3) nonprofit organizations, and (4) for-profit organizations. Health professionals were further divided into 2 subgroups—cardiologists and noncardiologists. This taxonomy facilitated the organization of videos by content similarity and differentiation.

### Evaluating Methodologies

The content, reliability, and quality of the videos underwent assessments via structured scoring protocols. We adopted 6 criteria from Goobie et al [28] to ascertain the comprehensive nature of the video content. These criteria scrutinized the extent to which videos covered the disease’s definition, signs and symptoms, risk factors, evaluation, management, and outcomes. Scoring for each facet used a 3-point scale: 0=unaddressed, 1=partially addressed, and 2=sufficiently addressed. A median score less than 1 for any category, or an overall median score under 6, indicated low content comprehensiveness. The DISCERN instrument was used to gauge content excellence, focusing on video reliability, treatment choices quality, and overall information quality (Multimedia Appendix 1) [29,30]. This instrument includes 16 items, scored on a 5-point scale (from 1=poor to 5=excellent). The initial 8 items assess the publication’s reliability, evaluating its clarity, relevance, balance, and fairness. This segment’s scores reflect the source’s trustworthiness in conveying specific disease treatments. The subsequent 7 items investigate treatment specifics, examining the presentation of each treatment’s effects and clarifying associated risks and benefits. Scores here represent the informational depth regarding treatment options, including self-management. The final query, based on previous elements, invites a summative assessment of the overall source quality concerning treatment information [24]. Median scores were 24 for publication’s reliability, 21 for treatment information quality, and 3 for overall quality, with an aggregate median DISCERN score of 48, designating scores below these as low quality. The DISCERN instrument, having been extensively validated, is prevalent in evaluating health content across various video-sharing platforms, including YouTube, TikTok, Kwai, and Bilibili [23,24,26,28,31]. Additionally, the Journal of the American Medical Association (JAMA) benchmark, assessing the credibility of video sources with a 0 to 4 scale (Multimedia Appendix 2) [32,33], was applied. This benchmark is divided into 4 categories, each meriting a point, with median scores of 2 or higher reflecting high quality, while lower scores indicate low quality. For the overall video quality assessment, the Global Quality Score (GQS) was used. This scale, ranging from 1=poor quality to 5=excellent quality, is widely recognized for evaluating web-based video content (Multimedia Appendix 3) [25,34,35]. Videos with scores of 4 or 5 were deemed high quality, those with a score of 3 were considered intermediate quality, and scores of 1 or 2 were classified as low quality.

### Evaluation Procedure

Two experienced cardiologists affiliated with a tertiary hospital, XG and MC, undertook the assessments. Their profound expertise in the field of cardiovascular medicine, augmented by specialized training, including cardiac surgery department rotations, positioned them as adept evaluators of specialized content. Consultations with the cardiac surgery team further refined the precision of their assessments. Video evaluations were conducted in a noninteractive manner, avoiding downloads, likes, comments, collections, or shares. The evaluators primed themselves by examining the American Heart Association and the European Society of Cardiology management guidelines [36-38] and acquainting themselves with the scoring instructions for DISCERN, JAMA, and GQS. Modifications were applied to tailor these methodologies for video content evaluation. Evaluations were carried out independently, with any divergences in ratings reconciled through collaborative dialogue. The team, having previously assessed CHD-related videos between March 2022 and July 2022, reached a consensus that enabled theoretical autonomy in evaluations. Nonetheless, to guarantee uniformity, preliminary discussions were compulsory prior to individual assessments. Following a consensus on the initial 20 videos, the evaluators independently reviewed the subsequent entries, subsequently computing the average scores.

### Statistical Analyses

Statistical assessments were executed using SPSS software (version 27.0, IBM Corp), while data visualization was achieved through GraphPad Prism software (version 9.0; Dotmatics). The consistency between raters was quantified by calculating Cohen κ coefficients, with values exceeding 0.75 indicating good interrater reliability. Comparative analyses of groups were conducted using the Kruskal-Wallis *H* test. Owing to the nonnormal distribution of certain datasets, Spearman correlation analysis was uniformly applied to evaluate the correlations between variable datasets. A threshold of *P<*.05 was established as the criterion for statistical significance.

## Results

### Video Characteristics

Upon the application of inclusion and exclusion criteria, we discerned a cohort of 145 videos eligible for subsequent data extraction and analysis (Figure 1). These videos were segregated into 4 classifications reflective of the uploader’s identity: health professionals, news agencies, nonprofit organizations, and for-profit organizations. As presented in Table 1, health professionals contributed 128 (88.3%) videos, news agencies accounted for 6 (4.1%) videos, nonprofit organizations for 10 (6.9%) videos, and for-profit organizations for 1 (0.7%) video. Within health professionals, cardiologists represented 117 (80.7%) entries, while noncardiologists constituted 11 (7.6%). The median time since upload was 323 (IQR 182-550) days; the median duration of the videos was 71 (IQR 52-125) seconds; and median indicators of engagement comprised 3372 (IQR 1223-17,000) likes, 159 (IQR 42-774) comments, 268 (IQR 65-1419) collections, and 578 (IQR 227-2433) shares. Notably, despite uniformity in video durations across sources, uploads by health professionals garnered more engagement metrics such as likes (median 5016, IQR 1537-22,000), comments (median 201, IQR 66-1064), collections (median 340, IQR 97-1808), and shares (median 893, IQR 271-2883) relative to other groups (Table 2).

**Table 1:** Proportion of videos by different types of uploaders.

Source	Description	Videos (n=145), n (%)
Health professionals	Individuals who describe themselves as health professionals	128 (88.3)
Cardiologists	Medical specialist who diagnoses, treats, and manages diseases and conditions related to the cardiovascular system	117 (80.7)
Noncardiologists	Medical specialist who is not specialized in cardiology	11 (7.6)
News agencies	Organizations providing news services	6 (4.1)
Nonprofit organizations	Organizations or hospitals operating in the public sector	10 (6.9)
For-profit organizations	Private sector organizations	1 (0.7)

**Table 2:** Video characteristics by different types of uploaders.

Source of videos	Days since upload, median (IQR)	Video duration (seconds), median (IQR)	Number of likes, median (IQR)	Number of comments, median (IQR)	Number of collections, median (IQR)	Number of shares, median (IQR)
Health professionals	285 (181-453)	72 (53-126)	5016 (1537-22,000)	201 (66-1064)	340 (97-1808)	893 (271-2883)
News agencies	875 (383-1086)	66 (45-112)	47 (10-1599)	1 (0-34)	5 (1-48)	21 (1-133)
Nonprofit organizations	406 (97-764)	68 (33-108)	280 (12-980)	6 (0-11)	6 (0-51)	35 (2-298)
For-profit organizations	817 (—^a^)	14 (—)	311 (—)	14 (—)	25 (—)	110 (—)
Overall	323 (182-550)	71 (52-125)	3372 (1223-17,000)	159 (42-774)	268 (65-1419)	578 (227-2433)

^a^Not applicable.

### Uploader TikTok Account Characteristics

Considering all videographers within the study, the median follower count was 563,000 (IQR 330,500-2,039,000), total likes on all submissions reached a median of 2,165,000 (IQR 916,000-15,439,500), and the median like quotient per video was 4716 (IQR 1454-24,504). Health professionals had the highest number of followers (median 629,000, IQR 385,000-2,039,000) and average likes per video (median 5686, IQR 1949-24,609; Table 3).

**Table 3:** TikTok account characteristics by different types of uploaders.

Source of videos	Followers, median (IQR)	Videos, median (IQR)	Total likes of all videos, median (IQR)	Average likes per video, median (IQR)
Health professionals	629,000 (385,000-2,039,000)	581 (258-757)	2,371,000 (1,107,000-17,471,000)	5686 (1949-24,609)
News agencies	446,000 (17,438-6,015,000)	1158 (415-11,420)	1,588,500 (117,633-114,996,250)	3262 (154-7543)
Nonprofit organizations	26,000 (9261-140,000)	453 (241-1049)	215,000 (95,750-701,250)	708 (252-1122)
For-profit organizations	818,000 (—^a^)	764 (—)	572,000 (—)	749 (—)
Overall	563,000 (330,500-2,039,000)	586 (263-772)	2,165,000 (916,000-15,439,500)	4716 (1454-24,504)

^a^Not applicable.

### Information Content Comprehensiveness

**Figure 2:**
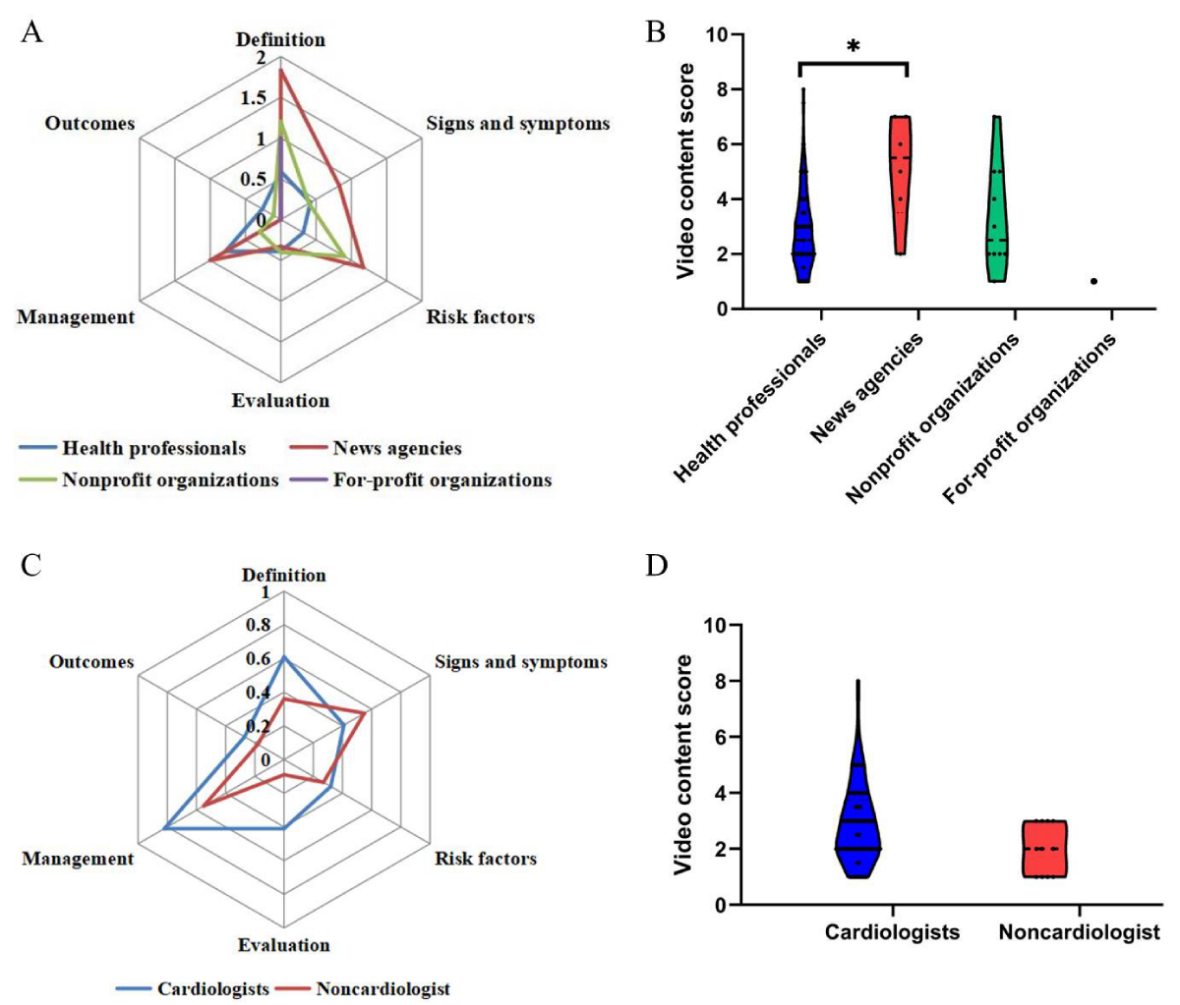
Comparison of comprehensiveness scores of video content among different types of uploaders. (A) Radar charts showing the scores of content comprehensiveness among videos from different types of uploaders. (B) Violin plots showing the total content comprehensiveness scores among videos from different types of uploaders. (C) Radar charts showing the scores of content comprehensiveness among videos from cardiologists and noncardiologists. (D) Violin plots showing the total content comprehensiveness scores among videos from cardiologists and noncardiologists.*P=.03.

The informational scope of the videos, encompassing 6 preestablished domains—definition, signs or symptoms, risk factors, evaluation, management, and outcomes—manifested varied levels of coverage as indicated in Table 4. In addition to a median score of 1 (IQR 0-1) for management, median scores for other domains were 0 (IQR 0-1), with a total score settling at 3 (IQR 2-4), as illustrated in Table 4 and Figure 2A. Overall, according to the methodological standards for content comprehensiveness, apart from the management scores, the scores for all other items did not reach the standard for high content comprehensiveness. Content comprehensiveness tallies attributed to news agencies markedly surpassed those of health professionals (*P*=.03), as portrayed in Figure 2B. Subgroup assessments among health professionals revealed that cardiologists’ median content comprehensiveness score was 3 (IQR 2-3.75), which nominally eclipsed that of their noncardiologist counterparts at 2 (IQR 1-3); however, this differential did not attain statistical significance (*P*=.051), as explicated in Table 4, along with Figures 2C and 2D.

**Table 4:** Comprehensiveness scores of video content by different types of uploaders.

Source of videos	Definition, median (IQR)	Signs and symptoms, median (IQR)	Risk factors, median (IQR)	Evaluation, median (IQR)	Management, median (IQR)	Outcomes, median (IQR)	Total, median (IQR)
Health professionals	0 (0-1)	0 (0-1)	0 (0-1)	0 (0-1)	1 (0-1)	0 (0-1)	2.5 (2-3.5)
Cardiologist	0 (0-1)	0 (0-1)	0 (0-1)	0 (0-1)	1 (0-1)	0 (0-1)	3 (2-3.75)
Noncardiologist	0 (0-1)	0 (0-1)	0 (0-1)	0 (0-0)	1 (0-1)	0 (0-0)	2 (1-3)
News agencies	2 (1.75-2)	1 (0-1.25)	1 (0.75-2)	0 (0-0.5)	1 (0.75-1.25)	0 (0-0)	5.5 (3.5-7)
Nonprofit organizations	1.5 (0-2)	0 (0-1)	1 (0-2)	0 (0-1)	0 (0-1)	0 (0-0)	2.5 (2-5)
For-profit organizations	1 (—^a^)	0 (—)	0 (—)	0 (—)	0 (—)	0 (—)	1 (—)
Overall	0 (0-1)	0 (0-1)	0 (0-1)	0 (0-1)	1 (0-1)	0 (0-0)	3 (2-4)

^a^Not applicable.

### Information Reliability and Quality

**Figure 3:**
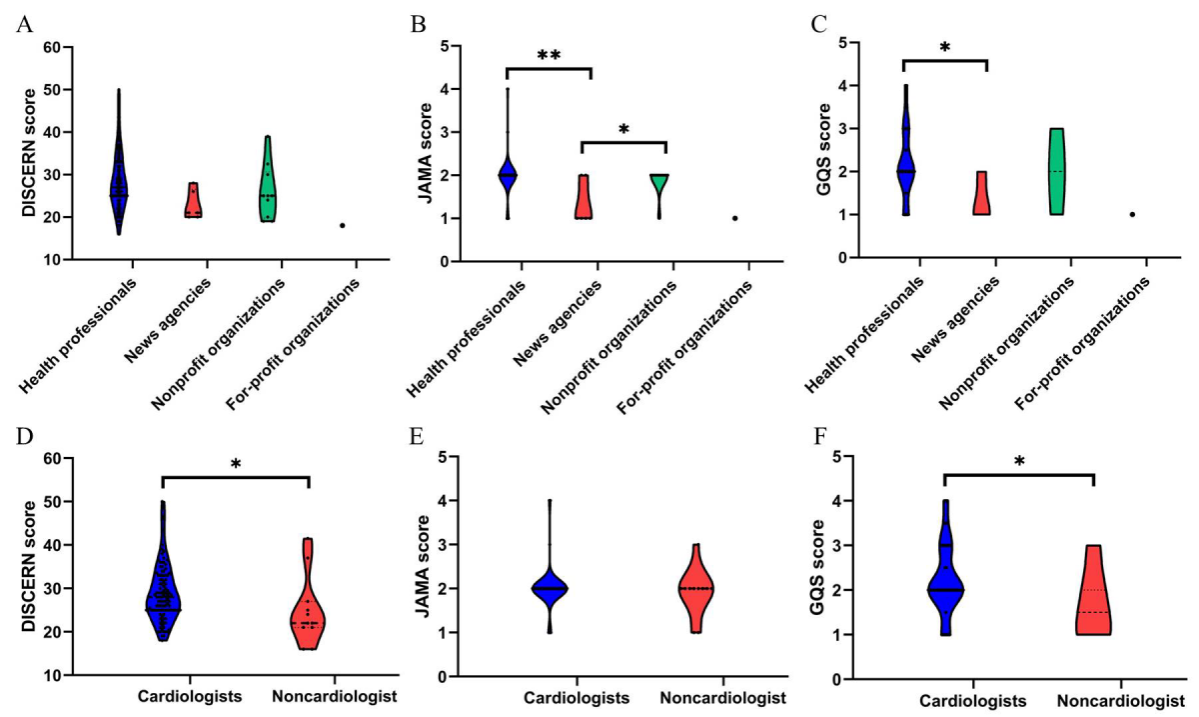
Comparison of quality scores of videos among different types of uploaders (DISCERN, JAMA, and GQS). (A) Violin plots showing the total DISCERN scores among videos from different types of uploaders. (B) Violin plots showing the total JAMA scores among videos from different types of uploaders. ** *P*<.001, * *P*=.02. (C) Violin plots showing the total GQS scores among videos from different types of uploaders. * *P*=.048. (D) Violin plots showing the total DISCERN scores among videos from cardiologists and noncardiologists. * *P*=.02. (E) Violin plots showing the total JAMA scores among videos from cardiologists and noncardiologists. (F) Violin plots showing the total GQS scores among videos from cardiologists and noncardiologists.* *P*=.02. GQS: Global Quality Score; JAMA: Journal of American Medical Association.

Regarding the reliability of video publications as assessed via the DISCERN instrument, the median score attributable to all submissions was 16 (IQR 14-19). In relation to the depicted treatment options’ quality within the videos, a median score of 8 (IQR 7-11) was observed for all entries. The overall quality and total scores were recorded at 2 (IQR 2-3) and 27 (IQR 24-32), respectively (Table 5). Subsequent assessment uncovered no notable discrepancy in overall scores amongst the varied groups (Figure 3A). Each video’s general quality was also assessed using the JAMA benchmarks and GQS. As indicated in Table 5, the median JAMA benchmark across all videos was 2 (IQR 2-2), and the median GQS score was 2 (IQR 2-3). Furthermore, the JAMA benchmarks for productions from health professionals and nonprofit organizations significantly exceeded those originating from news agencies (*P<*.001 and *P*=.02, respectively; Figure 3B). Additional analysis highlighted that the GQS indices for content curated by health professionals substantially outstripped those affiliated with news agencies (*P*=.048; Figure 3C). According to the methodological criteria for quality evaluation, the collective video materials failed to meet the median thresholds as stipulated by DISCERN and JAMA assessments, nor did they attain the high-quality benchmark of 4 points on the GQS . Consequently, the overall video quality is deemed low quality. Within the subgroup assessment, the median DISCERN score among cardiologists stood at 28 (IQR 25-33), surpassing the corresponding score for noncardiologists at 22 (IQR 21-27) and *P*=.02 (Figure 3D). For both cardiologist and noncardiologist groups, the median JAMA benchmarks remained constant at 2 (IQR 2-2), sans a discernible statistical variance (Figure 3E). The median GQS score for cardiologists, however, was evaluated at 2 (IQR 2-3), eclipsing that of their noncardiologist peers at 1.5 (IQR 1-2) and *P*=.02 (Figure 3F).

**Table 5:** Quality scores of videos by different types of uploaders (DISCERN, JAMA^a^, and GQS^b^).

Source of videos	DISCERN, median (IQR)					JAMA, median (IQR)		GQS, median (IQR)
	Publication reliability	Treatment choices	Overall quality	Total scores				
Health professionals	16 (14.63-19)	8.25 (7-11)	2 (2-3)	27 (25-33)	2 (2-2)		2 (2-3)	
Cardiologist	16 (15.25-19)	9 (7-11)	2 (2-3)	28 (25-33)	2 (2-2)		2 (2-3)	
Noncardiologist	13 (12.5-18)	7 (7-9)	1.5 (1-2)	22 (21-27)	2 (2-2)		1.5 (1-2)	
News agencies	13 (12-16.25)	7 (7-8.25)	1 (1-2)	21 (20-26.5)	1 (1-2)		1 (1-2)	
Nonprofit organizations	16 (11.75-17.75)	7 (7-8.38)	2 (1-3)	25 (19.75-30.63)	2 (2-2)		2 (1-3)	
For-profit organizations	10 (—^c^)	7 (—)	1 (—)	18 (—)	1 (—)		1 (—)	
Overall	16 (14-19)	8 (7-11)	2 (2-3)	27 (24-32)	2 (2-2)		2 (2-3)	

^a^JAMA: Journal of American Medical Association.

^b^GQS: Global Quality Scale.

^c^Not applicable.

### Correlation Analysis

The Spearman correlation analysis revealed certain correlations among the uploader account characteristics. Positive correlations were detected between DISCERN scores and follower count (*r*=0.296; *P<*.001), total likes (*r*=0.343; *P<*.001), and average likes (*r*=0.340; *P<*.001); JAMA benchmarks and follower count (*r*=0.191; *P*=.02), total likes (*r*=0.208; *P*=.01), and average likes (r=0.217; *P*=.009); and GQS scores and follower count (*r*=0.225; *P*=.007), total likes (*r*=0.295; *P<*.001), and average likes (*r*=0.305; *P<*.001; Table 6). The investigation further disclosed correlations germane to the video attributes themselves. Positive or negative correlations manifested between DISCERN scores and days since upload (*r*=–0.291; *P<*.001), video duration (*r*=0.598; *P<*.001), likes (*r*=0.432; *P<*.001), comments (*r*=0.434; *P<*.001), collections (*r*=0.425; *P<*.001), and shares (*r*=0.438; *P<*.001). Likewise, JAMA benchmarks showed positive or negative correlations with days since upload (*r*=–0.274; *P*=.001), likes (*r*=0.219; *P*=.008), comments (*r*=0.216; *P=*.009), and collections (*r*=0.200; *P*=.02). Finally, GQS scores exhibited positive or negative correlations with days since upload (*r*=–0.294; *P<*.001), video duration (*r*=0.572; *P<*.001), likes (*r*=0.347; *P<*.001), comments (*r*=0.359; *P<*.001), collections (*r*=0.332; *P<*.001), and shares (*r*=0.329; *P<*.001; Table 6).

**Table 6:** Correlations between uploader account and video characteristics with quality scores (DISCERN, JAMA^a^, and GQS^b^).

Variable and analysis			DISCERN				JAMA				GQS		
			*r* value		*P* value		*r* value		*P* value		*r* value		*P* value
**Uploader account**													
	Followers	0.296		<.001		0.191		.02		0.225		.007	
	Total likes of all videos	0.343		<.001		0.208		.01		0.295		<.001	
	Videos	–0.069		.41		–0.032		.70		–0.105		.21	
	Average likes	0.340		<.001		0.217		.009		0.305		<.001	
**Video**													
	Days since upload	–0.291		<.001		–0.274		.001		–0.294		<.001	
	Video duration	0.598		<.001		0.133		.11		0.572		<.001	
	Likes	0.432		<.001		0.219		.008		0.347		<.001	
	Comments	0.434		<.001		0.216		.009		0.359		<.001	
	Collections	0.425		<.001		0.200		.02		0.332		<.001	
	Shares	0.438		<.001		0.139		.10		0.329		<.001	

^a^JAMA: Journal of American Medical Association.

^b^GQS: Global Quality Scale.

## Discussion

### Principal Findings

In this comprehensive cross-sectional study, we analyzed the informational content of CHD-related videos on TikTok at a single time point, assessing their quality via the DISCERN, JAMA, and GQS instruments. Presently, TikTok enforces stringent authentication protocols to safeguard user interests and the integrity and dependability of information disseminated on its platform. Only certified individuals and organizations are permitted to post health-related videos on the TikTok platform. For individuals, this includes attending physicians or higher-ranked doctors at public tertiary hospitals and national master practitioners of medicine. As for organizations, it encompasses public hospitals rated secondary level and above, departments within public tertiary hospitals, social organizations, and medical media [39,40]. As a result, a predominant share of videos (128/145, 88.3%) originated from health professionals, with a mere single entry (n=1, 0.7%)—devoid of any promotional material—contributed by for-profit organizations, notably a medical educational establishment. These regulatory measures enhance the content's professional quality. Nevertheless, the anticipated quality benchmarks were not met, with notable disparities in quality ratings discerned across varied categories.

### Overall Quality of the Videos

Our findings underscore a lack of comprehensive coverage concerning all facets of CHD across the videos reviewed. Among the assorted video sources, news agencies were accorded the highest scores for content comprehensiveness. Moreover, in assessing the reliability and quality via the DISCERN, JAMA, and GQS instruments, most of the videos failed to receive high scores, culminating in an overarching assessment of low quality. Although DISCERN scores were uniformly distributed across groups, contributions from health professionals were appraised more favorably than those from news agencies in JAMA and GQS evaluations, and content from nonprofit organizations was rated more highly than that from news agencies in JAMA assessments. Within the health professionals, despite no discernible differences in content comprehensiveness and JAMA evaluations between cardiologists and noncardiologists, cardiologists demonstrated a significant superiority in DISCERN and GQS ratings.

### Correlations of Video Quality With Uploader Account and Video Characteristics

Metrics such as follower count, total likes across all videos, video count, and average likes per video can be indicative of an uploader’s operational level and popularity, whereas the volume of likes, comments, collections, and shares reflect the popularity of a video [41]. Our analysis identified positive correlations between follower count; total and average likes; and DISCERN, JAMA, or GQS scores, suggesting that uploaders of eminent operational level are predisposed to craft higher-quality videos. Pertaining to video specifics, positive correlations were established between likes, comments, collections, shares, and DISCERN, JAMA, or GQS evaluations, signifying that high-quality videos inherently possess a higher propensity for popularity. Intriguingly, the days since video upload inversely correlated with DISCERN, JAMA, and GQS evaluations, attributable to the progressive enhancement in video quality for content introduced at subsequent intervals [42]. Additionally, a direct correlation was observed between video duration and DISCERN or GQS evaluations, primarily as lengthier videos potentially furnish more comprehensive information, thereby augmenting quality [43].

### Social Media’s Potential in Improving Knowledge of CHD: Addressing the Gap Between Medical Resource Shortage and High Demands

The improvement of knowledge about CHD, its management, and treatment principles can lead to favorable disease outcomes and better health behaviors [44]. An enhanced understanding of risk factors for CHD is positively associated with adherence to various lifestyle modifications, including weight reduction, heightened physical activity, stress containment, and nutritional adjustments. Additionally, the attainment of lipid profile targets also bears a relationship with the breadth of general knowledge as well as the application of antihypertensive medication [45]. Such advancements necessitate implementation through efficacious health education; high-quality health education empowers patients by facilitating the comprehensibility of their condition, fostering collaborative relationships with health care practitioners, and endorsing self-directed care [26]. This proactive participation in their therapeutic regime amplifies disease cognizance and self-management competencies in individuals diagnosed with CHD, while concurrently mitigating recurrence risks and associated complications [46]. Contemporary research has substantiated the impact of appropriate education in diminishing corticosteroid use and psychological disquietude and enhancing self-management skills among patients with CHD [47]. Nevertheless, due to a paucity of medical resources, conventional hospital-based health education falls short of satisfying patient demand for health knowledge [6,7]. Despite these shortcomings within traditional hospital settings, the internet emerges as a pragmatic alternative [48]. TikTok, in particular, boasts several superiorities over institutional education, including dismissing temporal or locational constraints, low expenditure, enhanced privacy, and reduced distrust of a single information source. Thus, TikTok has ascended as a pivotal repository for patients seeking information on CHD. Therefore, we must thoroughly exploit the potential of social media exemplified by TikTok to bridge the gap between fulfilling patient knowledge needs for CHD and the insufficiency of medical resources.

### Improving Quality of Health-Related Videos on Social Platforms: Collaborations and Governance

While social media offers numerous advantages in health education, the enhancement of the quality of health-related videos on such platforms remains imperative. The content comprehensiveness, reliability, and quality of CHD knowledge transmission warrant improvement. First, despite current prohibitions against patient-submitted medical content on TikTok, policies ought to be drafted to catalyze patient immersion in the digital health domain and amplify care efficacy. Second, given the intricate taxonomy, diagnostic protocols, therapeutic modalities, and caregiving procedures endemic to CHD, professionals in cardiovascular health must command exhaustive expertise and relentlessly refine their diagnostic and therapeutic paradigms via ongoing training and engagement with contemporary literature and guidelines [49]. Nonetheless, a solitary cardiovascular practitioner is incapable of disseminating objective information within the confines of a brief video, constrained by personal resource limitations. Collaboration among a team [50] or support from video platforms themselves is indispensable to guarantee the creation of high-quality videos that bolster the veracity and trustworthiness of diagnostic and therapeutic data. Third, in consideration of the specialized and convoluted nature of medical content, video durations should be extended to incorporate comprehensive information without compromising the integrity of content quality. Fourth, for the creators, sculpting videos in harmony with the criteria of evaluative tools, notably adhering to the granular benchmarks set forth by DISCERN and JAMA, can elevate video quality, rendering them more digestible and trustworthy. The triumvirate of communicative tactics—verbal, vocal, and visual—could be harnessed in unison within the video, and if adeptly used, can significantly aid viewers in grasping the concepts or issues presented [51]. Moreover, heeding past scholars’ recommendations, when fashioning medical videos, creators might use the motivational framework of role modeling coupled with the health belief paradigm to engender amplified viewership and interaction [22]. Fifth, TikTok is encouraged to contemplate the inauguration of professional accreditation for video auteurs specializing in medical material. Such a certification mark would bolster audience discernment of authentic and proficient information, thus expediting its propagation [25]. The standard of CHD knowledge videos on TikTok is markedly inconsistent. This inquiry advocates for the selection of videos emanating from news agencies for comprehensive knowledge and gives precedence to submissions crafted by health professionals, especially cardiologists, for their dependability, clarity, and high quality. In addition, when selecting videos, one should weigh considerations such as the characteristics of the uploader account and the attributes of the video itself, favoring those with extended durations, more recent publication dates, better popularity, and emanating from highly regarded contributors.

### Strengths and Limitations

This study represents the first attempt to evaluate the quality of CHD-related videos on TikTok using multiple assessment tools (content comprehensiveness, DISCERN, JAMA, and GQS). Using a diverse array of assessment tools, each with a focus on distinct facets, facilitates multidimensional assessments of video quality, spanning the breadth of information coverage, publication quality, treatment option credibility, informational reliability, and overall quality. Moreover, our study delves into the correlations between video characteristics (likes, comments, collections, and shares) and video quality, as well as the correlations between uploader account profiles (followers, total likes of uploaded videos, total number of uploaded videos, and average likes per video) and video quality. It is through these correlation analyses that the robustness and applicative significance of our study’s outcomes are fortified. On one hand, the observed positive correlations between quality ratings and the popularity indicators of videos and uploaders diminish the probability of artificially manipulated metrics such as likes and comments. On the other hand, it furnishes viewers with criteria for selecting videos from specific sources. Nonetheless, several limitations warrant attention in the interpretation of our findings. First, our dataset was confined to videos disseminated on Chinese TikTok platforms, thereby constraining the extrapolation of our conclusions to other linguistic contexts (eg, English) and platforms (eg, YouTube). Second, the application of assessment tools like the DISCERN and JAMA benchmarks as methods for evaluating web-based videos has been subject to critique [51]. Looking ahead, future inquiries should embrace broader cross-linguistic comparative studies, using more suitable evaluation instruments to validate our findings.

### Future Directions

Web-based health promotion has attracted considerable attention, compelling TikTok to introduce measures that bolster the governance of medical and health-centric videos, thereby affirming their reliability and quality to some extent [52]. Yet, to our cognizance, no universal standards are expressly delineated for health-promotion videos at present. In light of the burgeoning popularity of video-sharing platforms, it becomes imperative to forge foundational benchmarks for content disseminated across these mediums. Such criteria should encompass multiple dimensions to navigate the crafting of medical videos, including (1) scientific accuracy of the content of the video; (2) clarity of the message given; (3) authority (creator); (4) pedagogy and educational basis; and (5) technical design including quality images and good visuals, production style, quality scripts, and clear sounds and no noises in the background [51]. Health care providers and platform administrators alike should spearhead efforts to rectify this lacuna. Despite the propensity for videos to render intricate data more digestible, lay viewers may grapple with deciphering professional terminology due to the intrinsic complexities of medical knowledge. To address this issue, health professionals ought to undergo instruction in producing videos that crystallize information while retaining an evidence-based foundation. Optimal health-promoting videos must achieve a balance among scientific accuracy, popularity, duration, and ease of understanding [26]. Beyond refining authentication protocols for content producers, video platforms should also enhance professional scrutiny mechanisms for submissions. Enabling disinterested parties to assess and rate videos—and rendering such assessments accessible to the audience—would be beneficial. This strategy would help viewers discern video quality and access accurate health information.

In the assessment and publication of such video content, additional considerations should be integrated: first, alongside evaluations by medical experts, the inclusion of layperson representatives of varying ages, educational levels, and medical knowledge is advisable for quality assessment. This strategy would illuminate the comprehension of CHD information by nonspecialists and the efficacy of content communication. Second, acknowledging that manual video quality assessments may involve certain biases and dampened efficiency, the rapid development of deep learning and artificial intelligence has showcased promising capabilities in informational appraisal. Future prospects could incorporate auxiliary infotech tools to mitigate biases and amplify efficiency. For instance, resorting to software for video preprocessing to curtail affiliation bias and streamline evaluation timelines, coupled with leveraging deep learning for the detection of spurious content [17,18]. With the swift advancement of artificial intelligence, methodologies such as machine learning and deep learning algorithms can probe user sentiments on social networks like TikTok [53], explore variations in emotive expression across different demographics, gauge the precision of micro-expression analysis [54], undertake comprehensive sentiment evaluations within the TikTok app [55], and identify the veracity of messages on social media [17,18]. This intimates that TikTok may eventually harness a constellation of algorithmic processes to discriminate the authenticity of medical video content and assess its quality. Third, given the potential for substantive shifts in TikTok’s algorithmic framework over time, periodic reassessment might become necessary for postsignificant algorithmic alterations to ensure the provision of recent findings and perspicacity.

### Conclusions

This study undertook an evaluation of the informational quality of 145 CHD-related videos disseminated on TikTok. The ensuing revelations indicated that the quality of these videos was inadequate and exhibited divergence contingent upon source types. Specifically, submissions from news agencies demonstrated superior content comprehensiveness, while those emanating from health professionals and nonprofit organizations manifested heightened standards of quality and dependability relative to news agencies. Within the health professionals, content crafted by cardiologists surpassed the quality of alternatives produced by noncardiologists. In light of TikTok’s escalating popularity, it becomes indispensable to encourage team collaboration and institute fundamental benchmarks to uplift the quality of medical videos, thereby unlocking TikTok’s immense potential for improving patient health knowledge.

## Appendix

Multimedia Appendix 1 DISCERN quality criteria for assessing the publication reliability and treatment choices quality of video (scoring ranges from 1 to 5).

Multimedia Appendix 2 The Journal of American Medical Association (JAMA) scoring (1 point for answer "yes" and 0 points for answer "no").

Multimedia Appendix 3 Global Quality Score (GQS; scoring ranges from 1 to 5).

## Abbreviations

CHD: coronary heart disease
GQS: Global Quality Scale
JAMA: Journal of American Medical Association

## Data Availability

The datasets generated and/or analyzed during this study are available from the corresponding author on reasonable request.
