# Supplementary material for: The Quality of Short Videos as a Source of Coronary Heart Disease Information on TikTok: Cross-Sectional Study
Source: JMIR Form Res. 2024 Sep 3;8:e51513. doi: 10.2196/51513 (PMC11408897; doi:10.2196/51513)
Supplement: Multimedia Appendix 1 [file formative_v8i1e51513_app1.docx]

| **Section 1 IS THE PUBLICATION RELIABLE?** | |
| --- | --- |
| 1 | Are the aims clear? |
| 2 | Does it achieve its aims? |
| 3 | Is it relevant? |
| 4 | Is it clear what sources of information were used to compile the publication (other than the author or producer)? |
| 5 | Is it clear when the information used or reported in the publication was produced? |
| 6 | Is it balanced and unbiased? |
| 7 | Does it provide details of additional sources of support and information? |
| 8 | Does it refer to areas of uncertainty? |
| **Section 2 HOW GOOD IS THE QUALITY OF INFORMATION ON TREATMENT CHOICES?** | |
| 9 | Does it describe how each treatment works? |
| 10 | Does it describe the benefits of each treatment? |
| 11 | Does it describe the risks of each treatment? |
| 12 | Does it describe what would happen if no treatment is used? |
| 13 | Does it describe how the treatment choices affect overall quality of life? |
| 14 | Is it clear that there may be more than one possible treatment choice? |
| 15 | Does it provide support for shared decision making? |
| **Section 3 OVERALL RATING OF THE PUBLICATION** | |
| 16 | Based on the answers to all of these questions, rate the overall quality of the publication as a source of information about treatment choices |
