# Supplementary material for: The Quality of Short Videos as a Source of Coronary Heart Disease Information on TikTok: Cross-Sectional Study
Source: JMIR Form Res. 2024 Sep 3;8:e51513. doi: 10.2196/51513 (PMC11408897; doi:10.2196/51513)
Supplement: Multimedia Appendix 2 [file formative_v8i1e51513_app2.docx]

| **Criteria** | **Description** |
| --- | --- |
| Authorship | Authors and contributors, their affiliations, and relevant credentials should be provided. |
| Attribution | References and sources for all content should be listed clearly, and all relevant copyright information noted. |
| Currency | Website ownership should be prominently and fully disclosed, as should any sponsorship, advertising, underwriting, commercial funding arrangements or support, or potential conflicts of interest. |
| Disclosure | Dates that content was posted and updated should be indicated. |
