# Supplementary material for: The Quality of Short Videos as a Source of Coronary Heart Disease Information on TikTok: Cross-Sectional Study
Source: JMIR Form Res. 2024 Sep 3;8:e51513. doi: 10.2196/51513 (PMC11408897; doi:10.2196/51513)
Supplement: Multimedia Appendix 3 [file formative_v8i1e51513_app3.docx]

| **GQS Definition** | **Score** |
| --- | --- |
| Poor quality, poor flow of the video, most information missing, not at all useful for patients | 1 |
| Generally poor quality and poor flow, some information listed but many important topics missing, of very limited use to patients | 2 |
| Moderate quality, some important information is adequately discussed | 3 |
| Good quality good flow, most relevant information is covered, useful for patients | 4 |
| Excellent quality and flow, very useful for patients | 5 |
